# Supplementary material for: SP142 PD-L1 Assays in Multiple Samples from the Same Patients with Early or Advanced Triple-Negative Breast Cancer
Source: Cancers (Basel). 2022 Jun 21;14(13):3042. doi: 10.3390/cancers14133042 (PMC9265054; doi:10.3390/cancers14133042)
Supplement: Supplementary file 1 [file cancers-14-03042-s001.zip › cancers-1748263-supplementary.pdf]

Table S1. Clinical and pathological characteristics of patients with early triple-negative breast cancer.

Variables	The Upfront Surgery Group (n = 47)	The NACT Group (n = 30)
Age (median, range)	58.0 (29–87)	47.9 (30–67)
Histologic type		
IDC	40 (85.1%)	28 (93.3%)
ILC	2 (4.3%)	0 (0%)
Others [a]	5 (10.6%)	2 (6.7%)
Clinical T stage, %		
T1	26 (55.3%)	3 (10%)
T2-4	21 (44.7%)	27 (90%)
Clinical N stage, %		
N0	45 (95.7%)	12 (40%)
N1-3	2 (4.3%)	18 (60%)
Histologic Grade in Biopsy, %		
Grade I	1 (2.1%)	0 (0%)
Grade II	35 (74.5%)	11 (36.7%)
Grade III	11 (23.4%)	19 (63.3%)
Pathologic T stage		
T1	31 (66%)	21 (70%)
T2-4	16 (34%)	9 (30%)
Pathologic N stage		
N0	41 (87.2%)	26 (86.7%)
N1-3	6 (12.8%)	4 (13.3%)
Histologic Grade in surgical specimen, %		
Grade I	0 (0%)	0 (0%)
Grade II	26 (55.3%)	15 (50%)
Grade III	21 (44.7%)	15 (50%)
TIL % at baseline at biopsy, (median, range)	25.43 (0–100)	33.55 (0–90)
TIL % at baseline at surgical sample, (median,range)	30.32 (0–100)	12.1 (0–50)
Ki67 labelling index (median, range)	41.62 (2.72–90.0)	43.15 (0–90)
Neoadjuvant chemotherapy regimen		
AC	-	2 (6.7%)
AC followed Taxane	-	26 (86.7%)
Others [b]	-	2 (6.7%)

a, upfront) Medullary 1, Cribrifrom 1, Metaplastic 1, Combined 2, NACT) Metaplastic 1, Combined 1. b. One is AC followed TC, the other is AC followed TP. Abbreviation: IDC, Invasive ductal carcinoma; ILC, Invasive lobular carcinoma; TILs, Tumor-infiltrating lymphocytes; AC, Anthracycline plus Cyclophosphamide; TC, Taxotere and Cyclophosphamide; TP, Paclitaxel plus Cisplatin.

Table S2. Metastatic TNBC patients who taken multiple PD-L1 test (*n* = 12).

Variables	Total patients (*n* = 12)
Age (Median, range)	45.7 (29–72)
Metastasis status	
recurrent and/or metastatic	11 (91.7%)
de novo stage IV	1 (8.3%)
Clinical T stage, %	
T1	4 (33.3%)
T2	6 (50.0%)
T4	2 (16.7%)
Clinical N stage, %	
N0	3 (25.0%)
N1-3	9 (75.0%)
Neoadjuvant CTx.	
Yes	8 (66.7%)
No	4 (33.3%)
Operation Method (N=12)	
BCS	5 (41.7%)
TM	6 (50.0%)
No surgery (de novo)	1 (8.3%)
RCB index (N=8)	
0	2 (25.0%)
1	0 (0%)
2	3 (37.5%)
3	3 (37.5%)
Adjuvant Chemotherapy	
Yes	8 (66.7%)
No [a]	4 (33.3%)
Metastasis area	
Locoregional	1 (8.3%)
Distant	7 (58.3%)
Locoregional with Distant	4 (33.3%)
Locoregional metastasis site (N=5)	
Ipsilateral breast	1 (20%)
Axilla, solitary	1 (20%)
Axilla, multiple	2 (40%)
Internal mammary node	1 (20%)
Distant metastasis site (N=11)	
Lung	5 (45.5%)
Liver	2 (18.2%)
Bone	2 (18.2%)
Brain	1 (9.1%)
Multiple	1 (9.1%)

a. including palliative chemotherapy (de novo). Abbreviation: TNBC, Triple-negative breast cancaer; CTx, Chemotherapy; BCS, Breast-conserving surgery; TM, Total mastectomy; RCB, Residual Cancer Burden.

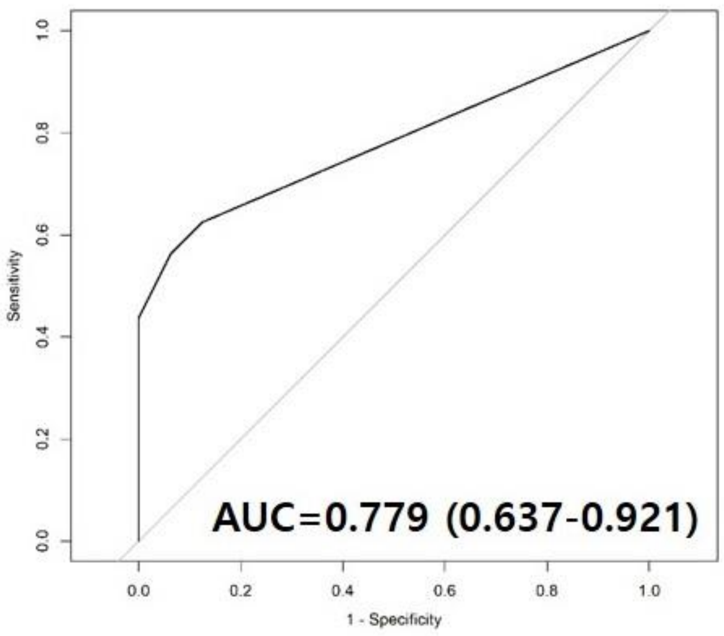

Figure S1. ROC curve predicting PD-L1 positive shift when 20% is set as the optimal cut-off of TIL. The AUC for the TIL score was 0.779 (95% CI 0.637-0.921). Abbreviation: ROC, Receiver operating characteristic; TIL, Tumor-infiltrating lymphocytes; AUC, Area under the curve
